# Maternal body mass index and country of birth in relation to the adverse outcomes of large for gestational age and gestational diabetes mellitus in a retrospective cohort of Australian pregnant women

**DOI:** 10.1186/s12884-021-04125-5

**Published:** 2021-09-23

**Authors:** Catherine R. Knight-Agarwal, Rati Jani, Meisa Al Foraih, Dionne Eckley, Carrie Ka Wai Lui, Shawn Somerset, Deborah Davis, Monica Yuri Takito

**Affiliations:** 1 Department of Nutrition and Dietetics, The University of Canberra, Locked bag 1, ACT Bruce, Australia; 2 Department of Human Movement, The University of São Paulo, São Paulo, Brazil

**Keywords:** Maternal obesity, Large for gestational age, Gestational diabetes mellitus

## Abstract

**Background:**

The prevalence of gestational diabetes mellitus in Australia has been rising in line with the increased incidence of maternal overweight and obesity. Women with gestational diabetes mellitus, high body mass index or both are at an elevated risk of birthing a large for gestational age infant. The aim was to explore the relationship between country of birth, maternal body mass index with large for gestational age, and gestational diabetes mellitus. In addition to provide additional information for clinicians when making a risk assessment for large for gestational age babies.

**Method:**

A retrospective cohort study of 27,814 women residing in Australia but born in other countries, who gave birth to a singleton infant between 2008 and 2017 was undertaken. Logistic regression analysis was used to examine the association between the aforementioned variables.

**Results:**

A significantly higher proportion of large for gestational age infants was born to overweight and obese women compared to those who were classified as underweight and healthy weight. Asian-born women residing in Australia, with a body mass index of ≥40 kg/m2, had an adjusted odds ratio of 9.926 (3.859–25.535) for birthing a large for gestational age infant. Conversely, Australian-born women with a body mass index of ≥40 kg/m2 had an adjusted odds ratio of 2.661 (2.256–3.139) for the same outcome. Women born in Australia were at high risk of birthing a large for gestational age infant in the presence of insulin-requiring gestational diabetes mellitus, but this risk was not significant for those with the diet-controlled type. Asian-born women did not present an elevated risk of birthing a large for gestational age infant, in either the diet controlled, or insulin requiring gestational diabetes mellitus groups.

**Conclusions:**

Women who are overweight or obese, and considering a pregnancy, are encouraged to seek culturally appropriate nutrition and weight management advice during the periconception period to reduce their risk of adverse outcomes.

## Statement of Significance

### Problem

Maternal body mass index (BMI) of ≥30 kg/m^2^ has been shown to increase the prevalence of pregnancy-related complications such as gestational diabetes mellitus (GDM) and large for gestational age (LGA), which in turn, may elevate future chronic disease risk for both women and offspring.

### What is already known

Migrants often have difficulty acculturating to their new country, which can have adverse consequences for dietary practices and weight management. In women of all ethnicities, maternal overweight and obesity increases the risk of GDM and LGA.

### What this paper adds

Both Asian and Australian born women with a body mass index of ≥30 kg/m^2^ (with or without GDM) had an increased risk of birthing an LGA infant compared to women of healthy weight. In the presence of insulin-requiring GDM women who were born in Australia were at increased risk of birthing an LGA infant, but insulin-requiring women with GDM born in Asia did not have this risk. Compared to those with no GDM, in the presence of diet-controlled GDM there was no increased risk of birthing an LGA infant for any women regardless of place of birth. Compared to Australian-born women, Asian-born women did not present an elevated risk of birthing an LGA infant, in either the diet-controlled or insulin-requiring GDM groups. Women should be supported to enter pregnancy with a healthy BMI. Culturally appropriate lifestyle interventions to prevent LGA and GDM are warranted.

## Background

In Australia, the prevalence of maternal overweight and obesity has been increasing largely in line with prevalence in the general population [1, 2] and indeed other industrialised countries [3, 4]. Between 2014 and 2015, data from the National Health Survey reported that there were over 2.5 million overweight and obese women residing in Australia (approximately 29% of all females aged 18 years and over) [1, 2]. Statistics from the nation’s capital of Canberra are comparable [1].

Numerous studies have shown that maternal pre-pregnancy BMI of ≥30 kg/m^2^ increases the prevalence of pregnancy complications, such as GDM and LGA, which can in turn elevate offspring risk for chronic disease in later life [5–11]. In addition, evidence exists that being born LGA is a predictor of obesity in adulthood [12]. The proportion of newborns with macrosomia, defined as having a birth weight of over 4 kg, ranges from less than 1.0–14.9% in developing countries to as high as 20% in northern Europe [10–12]. An Australian retrospective cohort study including 24,161singleton births between 2009 and 2015 demonstrated a significant association between overweight and obesity in early pregnancy and increased risks of preterm birth (PTB), LGA and admissions to neonatal intensive care units (NICU) [7]. Similar outcomes have been reported in Europe, the Middle East, North America and Asia [10, 13–19]. Several studies have also demonstrated a link between maternal obesity and insulin resistance, higher rates of type 2 diabetes mellitus and impaired adult cardiovascular health in adult offspring [20, 21]. Similar findings have been found in Australian cohorts [7].

A high proportion of the Australian population has been born in countries other than Australia [22]. In 2019, there were over 7.5 million immigrants living in Australia which is just under 30% of the total population. England continues to have the largest group of overseas-born individuals (3.9% of Australia’s total population). However, second and third place are China (2.7% of Australia’s total population) and India (2.6% of Australia’s total population), respectively. Median age of Chinese and Indian women immigrating to Australia is 34 years with the vast majority being childless but of child-bearing age [22]. This indicates that a significant number of babies born in Australia will be to parents who have migrated from Asia.

Previous studies have shown that migrants often have difficulty acculturating to their new country, which has consequences for dietary practices and other determinants of well-being [23]. The increased risk of chronic disease manifested in immigrants from low middle income countries (LMICs) is related in part to the transition from food environments of undernutrition, to overnutrition or both [23].

Given the high proportion of Australian women born elsewhere and their equally high rates of overweight and obesity [1, 22], they may be particularly vulnerable to the consequences of excessive body weight. This study, a secondary analysis of routinely collected clinical data, was undertaken to determine comparative risk of both Australian-born and Asian-born women regarding BMI, LGA and GDM. Such data is vital for informing the development of targeted interventions to maximize positive birthing outcomes particularly in women with high pre-pregnancy BMI’s and from various places of origin.

## Participants, ethics and methods

This retrospective cohort study was conducted using data from the Birthing Outcomes System (BOS) at a major tertiary institution in south eastern Australia between the 1st of January 2008 and the 31st of December 2017. There were 30,121 births over this period, with the hospital being the major maternity centre for a catchment population of 550,000. However, stillbirths and multiple pregnancies (i.e. twins, triplets) were excluded as were births where maternal BMI had not been recorded. This left 27,814 birth events for analysis. Ethical approval was obtained from the relevant Health Research Ethics and Governance Office. (no: ETHLR.18.048).

### Data assessment

Maternal BMI is calculated at the first antenatal appointment (usually at 12–14 weeks gestation) [24]. Classification of BMI was defined, according to WHO cut-offs, into four groups: underweight (< 18.5 kg/m^2^); healthy weight (18.5–24.9 kg/m^2^); overweight (25–29.9 kg/m^2^); obese class I (30–34.9 kg/m^2^); obese class II (35–39.9 kg/m^2^) and obese class III (> 40 kg/m^2^) [24].

Other demographic information which was collected included maternal age, maternal country of birth, relationship status, employment, smoking (both maternal and paternal), parity, and obstetric outcomes such as GDM, hypertensive disorders of pregnancy and premature rupture of membranes.

Maternal place of birth is recorded in the BOS database. Women were categorised into three broad groups: ‘all’(regardless of ethnicity) ‘Australian-born’ and ‘Asian-born’. The Standard Australian Classification of Countries (SACC), Second Edition [25] was used to define the nations to be included in this final category (for example China, India, Pakistan). It should be highlighted that the ethnicity of Australian-born women in our cohort is not readily available but comprises a mixture of predominantly European and Asian descent.

Gestational age was calculated from either the last menstrual period or the earliest ultrasound examination. The Australian national birthweight percentiles published by Dobbins et al., were used to calculate LGA defined as a birthweight >90th percentile for gestational age [26]. Birthweight results were expressed as SD (z) scores corrected for gestation at time of birth.

Maternity complications such as GDM and hypertensive disorders of pregnancy were defined according to the World Health Organisations (WHO’s) International Statistical Classification of Diseases and Related Health Problems manual [27]. Screening for GDM is universally conducted at the study hospital between 24- and 28-weeks’ gestation with a 75 g oral glucose tolerance test (OGTT). A positive diagnosis is made if the fasting plasma glucose is 5.1–6.9 mmol/L or if the 2-h post glucose load is 8.5–11.0 mmol/L [28]. Women with GDM receive group education from experienced dietitians and diabetes educators. This includes blood glucose monitoring, carbohydrate counting and recommended physical activity levels. Women are strongly encouraged to attend individual follow-up appointments either weekly or fortnightly in line with the Australasian Diabetes in Pregnancy Society (ADIPS) consensus guidelines for the testing and diagnosis of hyperglycaemia in pregnancy [28]. Data are entered into the database by clinicians contemporaneously or as soon as practicable after an episode of care with regular validation checks by the system administrator. Mandatory reporting fields are validated by the Epidemiology Section of the Department of Population Health at the jurisdiction level [29].

### Statistical analysis

Descriptive analysis was reported using means and standard deviations for continuous variables and frequencies and percentages for categorical variables. Binary logistic regression was performed, to assess the relationship between maternal BMI, GDM and LGA. Following this, multivariate binary logistic regression, using the forced entry method, was applied to associations found to be significant at the bivariate level. All models were adjusted for parity, baby gender, marital status, smoking, maternal country of birth, employment and premature rupture of membranes. These covariates are considered by clinicians working in the filed as important and have been used in similar published analyses on this topic [13–19, 30].

Cook’s distance values were used to examine for multivariate outliers and influential data points. All cases included in the study had Cook’s D values below one. No signs of multicollinearity were observed, and an acceptable goodness of fit model was found. Statistical significance was set at *p* < 0.05. Analyses were conducted using SPSS version 24 (SPSS Inc., Chicago, USA) [31].

## Results

The prevalence of GDM in the cohort was 10.9% and LGA 12.0%. Overall, a total of 27, 814 singleton birth events, with accompanying maternal BMIs, were included in the study (Table 1). Of these women: 1544 (5.6%) were underweight; 13,948 (50.0%) had normal BMIs; 6832 (24.6%) were overweight; 2967 (10.7%) were obese I; 1412 (5.1%) were obese II; and 1111 (4.0%) were obese III (Table 1).

**Table 1:** Frequency, percentage and unadjusted odds ratio’s for neonatal outcomes according to maternal and peripartum characteristics in a cohort of pregnant Canberran women, 2008–2017

	Total N	Large for gestational AGE n (%) OR crude (CI_95%_)	
**BMI (kg/m**^**2**^**)**			
< 18.5	1544	78 (5.1)	**0.559 (0.442; 0.707)**
18.5–24.9	13,948	1213 (8.7)	1
25–29.9	6832	955 (14.0)	**1.706 (1.559; 1.867)**
30–34.9	2967	534 (18.0)	**2.304 (2.063; 2.574)**
35–39.9	1412	293 (20.8)	**2.749 (2.386; 3.167)**
> 40.0	1111	279 (25.1)	**3.521 (3.037; 4.082)**
**Baby gender**			
Female	13,387	1517 (11.3)	1
Male	14,414	1835 (12.7)	1.141 (1.062; 1.227)
**Parity**			
0	12,256	1055 (8.6)	1
1	9602	1316 (13.7)	**1.686 (1.548; 1.837)**
2	3810	621 (16.3)	**2.067 (1.858; 2.330)**
3	1324	209 (15.8)	**1.990 (1.695; 2.337)**
> 4	820	150 (18.3)	**2.377 (1.970; 2.868)**
**Mother work**			
No	15,253	1854 (12.2)	1
Yes	12,561	1498 (11.9)	0.979 (0.910; 1.052)
**Country of birth**			
Australian	19,329	2659 (13.8)	1
Asian	5054	301 (6.0)	**0.397 (0.351; 0.449)**
Other*	3394	391 (11.5)	**0.816 (0.729; 0.914)**
**Women smokes during pregnancy**			
No	25,251	3139 (12.4)	1
Yes	2368	197 (8.3)	**0.639 (0.550; 0.743)**
**Partner smokes**			
No	23,916	2941 (12.3)	1
Yes	3898	411 (10.5)	**0.841 (0.754; 0.938)**
**Married or with a partner**			
No	3261	366 (11.2)	1
Yes	24,553	2986 (12.2)	1.095 (0.976; 1.229)
**Premature rupture of membrane**			
No	27,217	3297 (12.1)	1
Yes	597	55 (9.2)	**0.736 (0.557; 0.974)**
**PIHD**			
No	26,395	3138 (11.9)	1
Yes	1419	214 (15.1)	**1.316 (1.133; 1.529)**
**Gestational Diabetes (GDM)**			
No	24,793	2849 (11.5)	1
Yes	3021	503 (16.7)	**1.380 (1.239; 1.537)**

BMI: Body Mass Index, PIHD: Pregnancy-induced hypertension. Significant values are bolded

Neonatal outcomes included: 292 (1.0%) extremely low birthweight (< 1000 g); 235 (0.8%) very low birthweight (1000–1499-g); 1528 (5.5%) low birthweight (1500–2499 g); 22,541 (81.0%) normal (2500-3999); 2710 (9.7%) large (4000–4499 g); and 508 (1.8%) exceptionally large (4500 g and more). For gestational age there was: 265 (1.0%) of extreme prematurity (< 28 weeks); 2045 (7.4%) preterm (28–36.9 weeks); 25,248 (90.8%) born at term (37–41.9 weeks); and 256 (0.9%) born post-term (42 weeks and more).

There was no significant difference observed between underweight and healthy weight women in terms of the proportion of LGA babies. However, there was a significantly higher proportion of LGA neonates born to overweight and obese women compared to underweight and healthy weight women (Bonferroni, *p* < 0.001). In addition, we observed the trend of increasing maternal BMI with increasing incidence of LGA. Australian-born women with a BMI of ≥40 kg/m^2^ had an AOR of 2.661 (CI: 2.256; 3.139) for birthing an LGA infant. Furthermore, women born in Asia with a BMI of ≥40 kg/m^2^ were found to have an AOR of 9.926 (CI: 3.859; 25.535) for birthing an LGA infant (Table 2).

**Table 2:** Multivariate analysis of the association between LGA, GDM and maternal BMI by country of birth

		LGA		
		1st Model ^a^	2nd Model ^b^	3rd Model ^c^
		All Women	Only with woman born in Australia	Only with woman born in Asia
		(*n* = 27,708)	(*n* = 19,244)	(*n* = 5043)
	OR crude	OR adjusted		
	(CI_95%_)	(CI_95%_)		
**BMI (kg/m**^**2**^**)**				
< 18.5	0.559	0.604	0.497	0.968
	(0.442;0.707)	(0.476;0.767)	(0.368;0.670)	(0.591;1.584)
18.5–24.9	1	1	1	1
25–29.9	**1.706**	**1.598**	**1.584**	**1.552**
	**(1.559;1.867)**	**(1.457;1.752)**	**(1.426;1.760)**	**(1.170;2.058)**
30.0–34.9	**2.304**	**2.013**	**1.941**	**2.739**
	**(2.063;2.573)**	**(1.796;2.256)**	**(1.709;2.204)**	**(1.867;4.020)**
35–39.9	**2.749**	**2.28**	**2.144**	**3.727**
	**(2.386;3.167)**	**(1.968;2.641)**	**(1.826;2.517)**	**(1.978;7.021)**
≥40	**3.521**	**2.879**	**2.661**	**9.926**
	**(3.037;4.082)**	**(2.469;3.358)**	**(2.256; 3.139)**	**(3.859;25.535)**
**GDM**^**d**^				
No	1	1	1	1
Diet controlled	0.98	0.933	1.075	0.68
	(0.818;1.175)	(0.774;1.125)	(0.863;1.340)	(0.425;1.090)
Insulin controlled	**1.738**	**1.402**	**1.490**	1.335
	**(1.527;1.978)**	**(1.221; 1.609)**	**(1.266;1.754)**	(0.961;1.853)

Reference group is BMI 18.5–24.9 kg/m^2^. Significant results are bolded

^a^ 1st Model is adjusted by country of birth, parity, baby gender, maternal age (continuous) and variables presented at the table

^b^ 2nd Model is adjusted by parity, baby gender, smoking, country of mother birth, work and premature rupture of membrane

^c^ 3rd Model is adjusted by parity, baby gender, smoking, country of mother birth, work and premature rupture of membrane

^d^ Reference group is no gestational diabetes

BMI - Body Mass Index; LGA - Large for Gestational Age; GDM - Gestational Diabetes Mellitus

Australian-born women with insulin-requiring GDM,, were found to have a significantly higher risk for birthing an LGA infant compared to Asian-born women who also had insulin-requiring GDM. Compared to women with either no GDM or insulin-requiring GDM, those with diet-controlled GDM, regardless of country of origin, did not demonstrate a significant increased risk for LGA.

## Discussion

In our study both Australian and Asian-born women who were overweight or obese had a significantly elevated risk of birthing an LGA infant when compared to their healthy weight counterparts. Our results show that Asian-born women with a BMI of > 40 kg/m^2^ were almost 10 times more likely to birth an LGA infant compared to Asian-born women within a healthy weight range despite adjusting for covariates such as parity and maternal age. A recent US population-based cohort study of 2,842,278 singleton births reported the odds of having an LGA baby was greatest for obese Asian Americans with an AOR of 2.05 (CI:1.91, 2.20) compared to other racial/ethnic groups in the same class of BMI [32].

A recent retrospective study undertaken with an Australian maternity cohort (*n* = 73, 517) reported that Chinese-born women had a 4-fold higher risk of GDM, despite having a lower pre-pregnancy BMI, than Caucasian women. Interestingly, after adjusting for confounders, Chinese-born women with GDM had a lower risk of birthing an LGA infant compared to their Caucasian counterparts. The authors suggested this may be related to physiological differences, migration patterns, and GDM experiences in a sociocultural context [33]. A Canadian retrospective cohort study was undertaken to determine the association between Chinese or South Asian (Indian) ethnicity and adverse neonatal and maternal outcomes for women with GDM compared to the general population. In contrast to infants of women from the general population (55.5%), infants of Chinese mothers had a lower risk of an adverse outcome at birth (42.9%, AOR 0.63, CI: 0.58–0.68), whereas infants of South Asian (Indian) mothers had a higher risk (58.9%, AOR 1.15, CI: 1.07–1.23). Clearly, the likelihood of GDM complications differed significantly between Chinese-born and Indian-born women and the general population [34]. Despite us observing an upward trend for LGA in Asian-born women with insulin-requiring GDM this finding did not reach statistical significance. A possible explanation may be the low numbers in the cohort or, indeed, there may be a real difference between the two populations. Nevertheless, further studies are recommended.

Another retrospective cohort study conducted by Yang and colleagues revealed that two separate variables, namely pre-pregnancy overweight and GDM, were both associated with an increased risk of LGA and that they also displayed a synergistic effect on its occurrence [35]. In their analysis, they adjusted for treatment modes such as insulin therapy or diet-control only, in order to minimize possible confounding effects which could result from different types of GDM management. The authors found co-existence of high BMI and GDM predisposed women residing in China (and of Chinese ethnicity) to a 5-fold risk of LGA when compared with women who had a BMI within the healthy range with no GDM [35]. Likewise, we demonstrated in our analysis that risk for LGA in Asian born women increased with increasing BMI. Even though women born in Asia demonstrated an elevated risk of LGA in the presence of insulin-requiring GDM this finding was not statistically significant.

Pedersen’s hypothesis states that elevated levels of maternal blood glucose traverse the placenta, but maternal insulin does not [36]. As a result, the pancreatic islet cells of the fetus are stimulated to secrete insulin, leading to fetal hyperinsulinemia. A consequence of this process is excessive accumulation of fetal adipose tissue leading to an increase in body weight. In addition, it is widely recognised that high maternal BMI can aggravate offspring obesity through genetic predisposition and the in-utero environment [10]. Such metabolic derangements could be a plausible explanation as to why, within our cohort, both Australian-born and Asian-born women with diet-controlled GDM did not display a statistically significant higher risk of LGA when compared to their non-GDM counterparts. Mild hyperglycaemia is likely to be controlled by dietary measures, especially in women who are highly motivated. In fact, those with diet-controlled GDM in our cohort appear to be at lower risk of LGA compared to those with either insulin-requiring GDM or no GDM however this trend was not statistically significant.

There are limitations to our study that should be acknowledged. Use of a single Asian-born group may not provide an accurate representation of the outcomes reported for LGA and GDM. There may be considerable heterogeneity, among Asian subgroups, highlighting the importance of disaggregation to assess ethnic differences. We did not have access to information on the gestational week when OGTT was performed, the degree of glycaemic control achieved by women or ethnic differences in adherence with GDM treatment. Another acknowledged limitation of this study is that some women who were deemed to have GDM under the old ADIPS criteria (pre-2013) would not have been identified as having GDM under the more recent criteria and vice-versa [28]. Future analyses should consider examining pre 2013 and post 2013 GDM cohorts separately. Detailed information on nutrition, physical activity, lack of resources and gestational weight gain (GWG), which may be factors underlying the racial differences in LGA risk, were unavailable [37]. In addition, ethnicity-specific cut-off points are not routinely used at the study hospital for the calculation of maternal BMI because this study looks at country of birth not ethnicity. Nevertheless, country of birth was controlled for in our analyses. BMI documentation is entered into the birthing outcomes system database, occurring at the first antenatal visit, typically around 14–16 weeks. However, there is evidence in the literature that most women put little (if any) weight on during their first trimester of pregnancy. In a cross-sectional analysis of 1000 women (gestational age) there was no reported change in mean maternal weight, BMI, total body water, fat mass, fat-free mass or bone mass before 14 weeks gestation [38].

It would be interesting to see if our results differed using birth weight percentiles for each of the racial groups identified in our cohort. Finally, we know from previous published literature, that obesity increases risk of GDM and GDM increases risk of LGA [10, 11]. However, we were unable to confirm the inter-relationship between these two variables for increased risk of LGA. Future analysis (using this dataset) could involve mediation analysis between all three variables.

While Asian-born women residing in Australia are eligible to access the same health services as women born in Australia, they may experience several challenges including connecting with antenatal care services, insufficient support, English language difficulties and transport issues. From a clinical perspective, maternal BMI, country of birth, LGA and GDM are important factors in the provision of culturally appropriate interventions in order to improve outcomes.

## Conclusion

Because high maternal BMI is a risk factor for GDM, decreasing the prevalence of overweight and obesity should reduce the incidence of both GDM and LGA. Both Australian and Asian-born women who were overweight or obese have a significantly elevated risk of birthing an LGA infant when compared to their healthy weight counterparts. Women with insulin requiring GDM born in Australia have a higher risk of LGA babies than Asian born women with the same condition. This information can assist clinicians in assessing risk both prior to pregnancy and early in antenatal care.

## Data Availability

The dataset analysed during the current study is not publicly available due to ethical approval requirements but is available from the corresponding author on reasonable request.

## Abbreviations

BOS: Birthing Outcomes Systems
BMI: Body Mass Index
LGA: Large for Gestational Age
GDM: Gestational Diabetes Mellitus
PIHD: Pregnancy-induced Hypertensive Disorder
HBW: High Birth Weight
HIC: High- income Countries
